# Protective Effects of Green Tea Supplementation against Lead-Induced Neurotoxicity in Mice

**DOI:** 10.3390/molecules27030993

**Published:** 2022-02-01

**Authors:** Areej Al-Qahtani, Jamaan Ajarem, Mohammad K. Okla, Samina Rubnawaz, Saud A. Alamri, Wahidah H. Al-Qahtani, Ahmad R. Al-Himaidi, Hamada Abd Elgawad, Nosheen Akhtar, Saleh N. Maodaa, Mostafa A. Abdel-Maksoud

**Affiliations:** 1 Department of Botany and Microbiology, College of Science, King Saud University, Riyadh 11451, Saudi Arabia; aamalkahtanee@kau.edu.sa (A.A.-Q.); saualamri@ksu.edu.sa (S.A.A.); 2 Department of Zoology, College of Science, King Saud University, Riyadh 11451, Saudi Arabia; jaarem@ksu.edu.sa (J.A.); ahimaidi@ksu.edu.sa (A.R.A.-H.); maodaa_28@yahoo.com (S.N.M.); harrany@gmail.com (M.A.A.-M.); 3 Department of Biochemistry, Faculty of Biological Sciences, Quaid I Azam University, Islamabad 45320, Pakistan; 4 Department of Food Sciences & Nutrition, College of Food & Agriculture Sciences, King Saud University, Riyadh 11451, Saudi Arabia; wahida@ksu.edu.sa; 5 Integrated Molecular Plant Physiology Research, Department of Biology, University of Antwerp, 2020 Antwerpen, Belgium; hamada.abdelgawad@uantwerpen.be; 6 Department of Biological Sciences, National University of Medical Sciences, Rawalpindi 46000, Pakistan; nosheenakhtar@numspak.edu.pk

**Keywords:** caffeine, dyslipidemia, GC/MS, lead toxicity, neurobehavior

## Abstract

The use of natural products as therapeutic agents is rapidly growing recently. In the current study, we investigated the protective effects of green tea supplementation on lead-induced toxicity in mice. Forty albino mice were divided into four groups as follows: A: control group; B: green tea receiving group; C: lead-intoxicated group; and D: lead-intoxicated group supplemented with green tea. At the end of the experiment, the animals were tested for neurobehavioral and biochemical alterations. Green tea was analyzed through Gas Chromatography–Mass Spectrometry (GC/MS) analysis. We found that supplementation with green tea ameliorated the lead-associated increase in body weight and blood glucose. Green tea supplementation also changed the blood picture that was affected due to lead toxicity and ameliorated lead-induced dyslipidemia. The group of mice that were supplemented with green tea has shown positive alterations in locomotory, anxiety, memory, and learning behaviors. The GC/MS analysis revealed many active ingredients among which the two most abundant were caffeine and 1,2-benzenedicarboxylic acid, mono(2-ethylhexyl) ester. We concluded that green tea supplementation has several positive effects on the lead-induced neurotoxicity in mice and that these effects may be attributed to its main two active ingredients.

## 1. Introduction

Lead is a chemical toxicant, which can cause severe damage to the blood [1] and many body organs like the liver, kidney, brain, spleen, and lungs [2]. It is one of the most important toxic heavy elements in the environment [3] which can penetrate the blood–brain barrier (BBB) and hence results in lead poisoning that can cause non-traumatic brain injury (NTBI) [4]. Not only does it break through the BBB, but it also increases its permeability, thus leaving the brain vulnerable to other toxic substances [5]. In humans, lead poisoning can lead to persistent cognitive deficit, which is more disabling than the physical complications [6]. In such cases, comprehensive neuropsychological testing and cognitive rehabilitation are essential in the treatment of lead poisoning [7]. In mice models, lead toxicity has several pathological consequences as liver injury [8] and brain damage [9].

The search for new safe natural therapeutic agents is now attracting many research groups globally [10]. Many natural compounds have revealed positive effects against different pathological conditions [10,11]. Green tea (*Camellia sinensis*) is a popular and commonly consumed drink, and its extract is found in many herbal and dietary supplements [12]. The protective effects of green tea and its main constituents against natural and chemical toxins are now being extensively investigated [13]. Green tea improved some neuro-functional and biochemical signs of arsenic toxicity in rats [14]. The protective effect of green tea on lead-induced oxidative and DNA damage in rat blood and brain tissue homogenates has also been demonstrated [15,16]. It can also protect against lead toxicity in rat kidneys [17], livers [18], and brains [19]. Human studies have provided evidence of an association between lead concentrations in blood and green tea consumption [20]. This may explain the reason why natural products, micronutrients, and nutraceuticals are now being used to treat depression [21,22]. The current study aimed to investigate the positive effects of green tea on the physiological and neurobehavioral changes induced by lead intoxication in mice.

## 2. Results

### 2.1. Ameliorating Effect of Green Tea Intake on Body Weight in Lead-Intoxicated Mice

As illustrated in Table 1, the group of mice that have been supplemented with green tea has shown no significant change in the body weight (33.29 ± 2.42) as compared to the control group (34.30 ± 1.72). On the other hand, a significant increase (*p* < 0.05) in body weight has been observed in the lead-intoxicated group (37.9 ± 1.07) in comparison to the control group (34.30 ± 1.72). Surprisingly, the lead-intoxicated group that has been supplemented with green tea, has shown restoration in the bodyweight increase (35.19 ± 1.14) as compared to the lead-intoxicated group (37.9 ± 1.07). Water consumption was decreased in both of lead intoxicated group (10.02 ± 0.17) and the green tea supplemented-lead intoxicated group (12.10 ± 0.16) as compared to the control (13.75 ± 0.15). Meanwhile, no significant change could be detected between the green tea supplemented group (14.49 ± 0.34) and the control group (13.75 ± 0.15).

### 2.2. Green Tea Supplementation Has Improved Hematological Parameters in the Lead-Intoxicated Mice

The effect of green tea supplementation on the cell blood count (CBC) was investigated in the experimental groups. As illustrated in Table 2, the green tea supplemented group has shown a CBC resembling that of the control with slight but no significant change in the red blood corpuscles (RBCs) count, white blood corpuscles (WBCs) count, hemoglobin (Hb) content, hematocrit (HCT), mean corpuscular volume (MCV), mean corpuscular hemoglobin (MCH), mean corpuscular hemoglobin concentration (MCHC), and platelets (PLT) count. Conversely, the lead intoxicated group of mice has shown a disturbed CBC characterized by a significant increase (*p* < 0.05) in the number of WBCs (9.34 ± 1.55), HCT (49.60 ± 1.33), and PLT (793 ± 78.37). In the lead-intoxicated group that has been supplemented with green tea (Lead + GT group), the green tea supplementation ameliorated the lead-associated hematological abnormalities to some extent. Here, the number of RBCs (8.16 ± 0.33), WBCs (8.03 ± 1.28), HCT (43.86 ± 1.62), MCV (53.80 ± 0.66), and PLT (685.6 ± 23.8) has been reached to near control values when compared to the lead intoxicated group.

### 2.3. Hypoglycemic Effect of Green Tea Supplementation against Elevated Blood Glucose Levels in Mice

**Figure 1:**
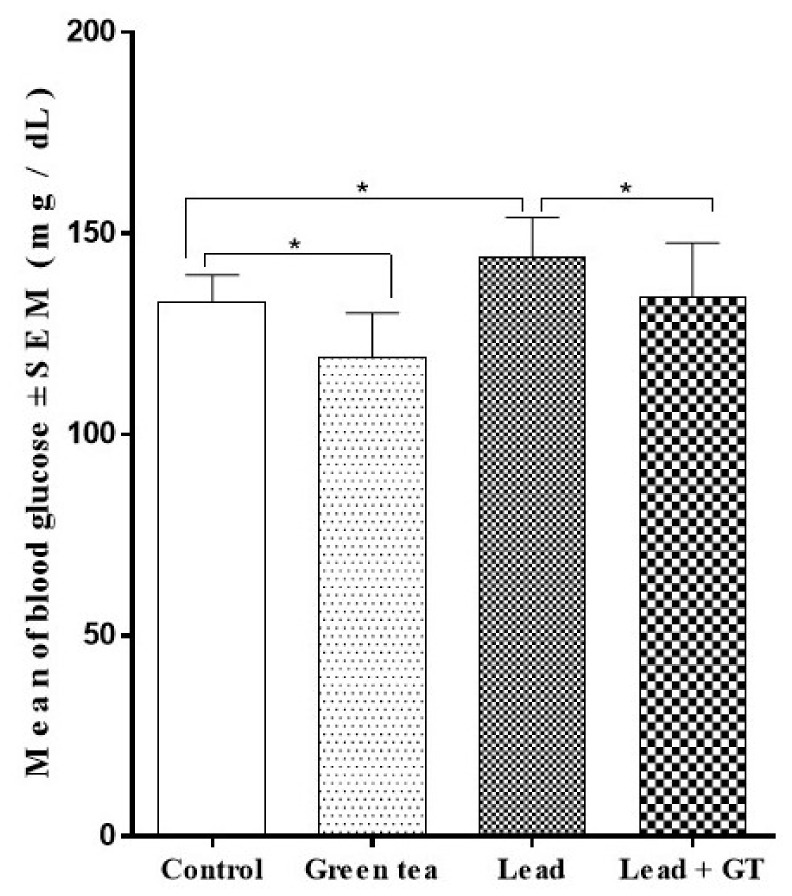
Effect of green tea supplementation on the blood glucose (mg/dL) level. Values are represented as Mean ± SEM., n = 10. * = *p* < 0.05.

As illustrated in Figure 1, the supplementation with green tea has a hypoglycemic effect represented by a decreased blood glucose level (119.20 ± 11.00) in the group of mice receiving green tea only as compared to the control group (132.94 ± 6.72). Lead intoxication has increased blood glucose levels (144.12 ± 9.82) in comparison to the control. However, the oral supplementation with green tea in the lead-intoxicated group has exerted a significant blood-glucose-lowering effect (*p* < 0.05) compared to the lead intoxicated group. Moreover, this group (Lead + GT) also presented glucose values comparable to the normal control group. Overall, the glucose levels followed the pattern of Lead > Lead + GT > Control > Green tea among different mice groups.

### 2.4. Ameliorating Effect of Green Tea on the Lead Intoxication-Associated Dyslipidemia

**Figure 2:**
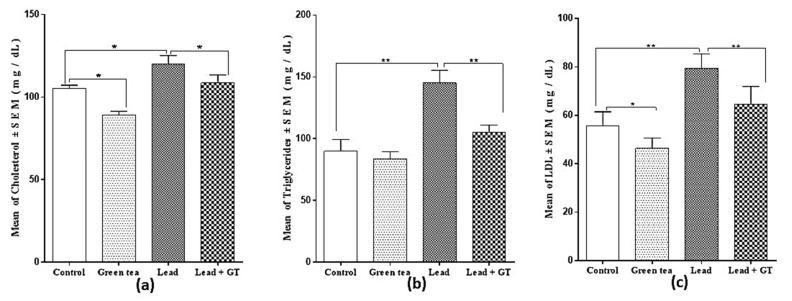
(**a**) Effect of green tea supplementation on the cholesterol level. (**b**) Effect of green tea supplementation on the triglycerides level. (**c**) Effect of green tea supplementation on the low-density lipoprotein (LDL) level. Mean ± SEM., n = 10. * = *p* < 0.05, ** = *p* < 0.01.

The effect of green tea on the blood levels of cholesterol, triglycerides, and LDL was also investigated. The green tea supplemented group of animals has lower levels of cholesterol (89.20 ± 2.27) (Figure 2a), triglycerides (83.52 ± 5.99) (Figure 2b), and LDL (46.32 ± 4.32) (Figure 2c) compared to the control group. The lead-intoxicated group has shown a dyslipidemic profile represented as increased blood levels of cholesterol (114.00 ± 9.12), triglycerides (145.20 ± 10.18), and LDL (79.30 ± 9.09) compared to the control group. However, the lead-intoxicated group supplemented with green tea has shown an amelioration in the lipid profile as represented by the restoration of near-normal values of blood levels of cholesterol (108.60 ± 7.86), triglycerides (104.96 ± 6.12), and LDL (64.54 ± 7.40) compared to the control group. Overall, Lead + GT group showed a more significant decrease (*p* < 0.01) in the triglyceride and LDL content than the cholesterol level (*p* < 0.01), which is initially increased by lead poisoning.

### 2.5. Decreased Accumulation of Lead in Blood and Brain of Green Tea-Supplemented Mice

**Figure 3:**
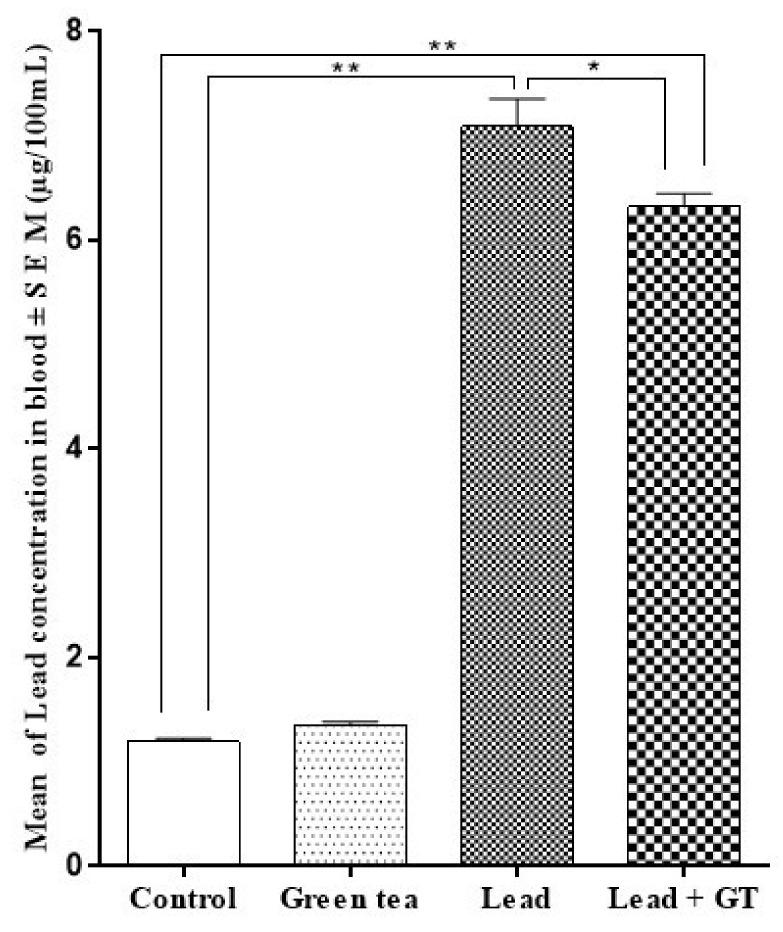
Effect of green tea supplementation on the lead concentration in blood. Mean ± SEM., n = 10. * = *p* < 0.05, ** = *p* < 0.01.

The concentration of lead in both blood and brain was estimated. As illustrated in Figure 3, no significant change in the blood level of lead could be detected between both control (1.20 ± 0.026) and green tea supplemented (1.36 ± 0.028) groups. Conversely, a highly elevated (*p* < 0.01) level of lead (7.09 ± 0.266) was found in the blood samples from the lead-intoxicated group of mice. The supplementation with green tea has significantly decreased (*p* < 0.05) this elevated level of lead in blood (6.32 ± 0.128). However, this level is still significantly higher (*p* < 0.01) compared to either the control group (1.20 ± 0.026) or the green tea-supplemented group (1.36 ± 0.028).

**Figure 4:**
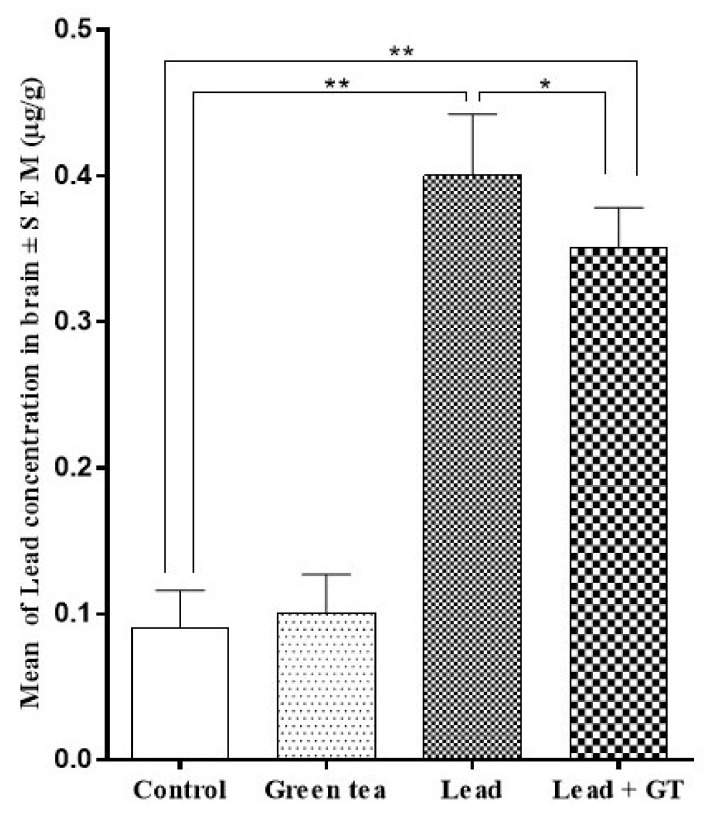
Effect of green tea supplementation on the lead concentration in the brain. Mean ± SEM., n = 10. * = *p* < 0.05, ** = *p* < 0.01.

On the other hand, the cerebral concentration of lead (Figure 4) in the lead-intoxicated group (0.40 ± 0.172) has been significantly elevated (*p* < 0.01) in comparison to both control (0.09 ± 0.026) and the green tea (0.10 ± 0.027) groups of animals. Similar to the situation in blood, green tea supplementation significantly decreased (*p* < 0.01) the cerebral lead concentration. Additionally, the lead-intoxicated group of animals that have received the green tea had a significantly elevated level (*p* < 0.01) of lead (0.35 ± 0.128) in comparison to the control (0.09 ± 0.026).

### 2.6. Ameliorating Effect of Green Tea on the Locomotory Behavior of Lead-Intoxicated Mice

When investigating the locomotory behavior of the green tea supplemented group in the open area, all the number of squares-crossed, the number of rears, the number of wall-rears, and the locomotion duration have been increased compared to the control group (Table 3). The immobility duration has decreased while the number of washings was not affected. Conversely, in the lead-intoxicated group, the locomotory behavior was negatively affected. All of the number of squares-crossed, the number of rears, the number of wall-rears, and the locomotion duration have been decreased in comparison to the control group. The immobility duration and the number of washings have been increased. Green tea supplementation has positively affected the lead-associated disturbed behavioral responses. The recorded values of the number of squares-crossed, the number of rears, the number of wall-rears, and the locomotion duration have indicated ameliorating effect which sometimes may reach to near the control group values.

### 2.7. Antianxiety Effect of Green Tea Supplementation on the Lead-Intoxicated Mice in the Plus-Maze

The anxiety behavior of all experimental groups of mice was analyzed with the aid of a plus-maze. In the green tea supplemented group, the number of entries into the open arm has been increased (9 ± 0.92) while that of entries into closed-arm has decreased (4.5 ± 1.09) when compared with the control group. Similarly, the time spent in the open arm has increased (173 ± 0.19) while the time spent in the closed arm (83 ± 0.24) and the maze center (38 ± 0.34) has decreased. Oppositely, the lead-intoxicated group have shown a greater anxiety as represented by the decrease in the number of entries into the open arm (6.8 ± 0.64), the increase in the number of entries into the closed arm (6.5 ± 0.66), and the similar disturbance in the time spent in the open arm (60 ± 0.2), closed arm (150 ± 0.23), and in the maze center (57 ± 0.29) compared to the control (Table 4). Green tea supplementation has a clear positive effect on this behavior as represented by the number of entries into the open arm (7.1 ± 0.81), that of entries into the closed arm (5.6 ± 1.78), and the time spent in the open arm (129 ± 0.19), in the closed arm (110 ± 0.27) and the maze center (48 ± 0.16).

### 2.8. Neuroprotective Effects of Green Tea Supplementation as Indicated from Learning and Memory Testing in Automatic Reflex Conditioner

Learning and memory responses were tested in all groups using the automatic reflex conditioner (shuttle box). In the green tea supplemented group, the total latency time (La) and the number of reinforced crossings (Re) during the shock have decreased while the number of intertrial crossing (Ic), the number of the crossing during light and sound stimulus (St) and the number of no crossing during the shock (Tr) have increased compared to the control group (Table 5). The lead-intoxicated group has shown an opposite response, whereas La and the Re have increased while Ic, St, and Tr have decreased compared to the control group. Supplementation with green tea has improved the lead-associated disturbance in learning and memory behaviors. Both La and Re have increased compared to the control, while Ic, St, and Tr have decreased compared to the control group.

### 2.9. GC/MS Analysis

**Figure 5:**
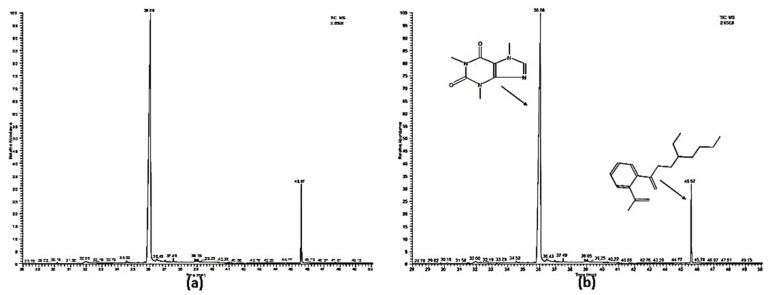
Chromatograms for the (**a**) aqueous extracts and (**b**) the ethanolic extract of green tea. (x-axis = Retention time; y-axis = % intensity/% abundance).

GC/MS analysis for both aqueous and ethanolic extracts of green tea has revealed two important active ingredients as illustrated in the chromatograms (Figure 5a,b). The first one was the caffeine that appeared at retention time (t_R_ 36.06 min) and the second one was 1,2-benzenedicarboxylic acid, mono(2-ethylhexyl) ester that appeared at t_R_45.57 min. Besides these two compounds, other compounds have appeared at different retention times with relatively small peaks. Surprisingly, both the aqueous and the ethanolic extracts have completely matched their chromatograms.

**Figure 6:**
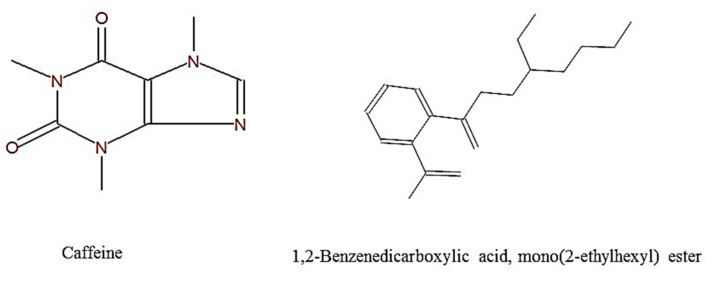
Chemical structure of caffeine and 1,2-benzenedicarboxylic acid, mono(2-ethylhexyl) ester.

The cyclic structure of both compounds is illustrated below in Figure 6.

## 3. Discussion

Green tea is a popular natural beverage used around the world. The current study investigated the positive effects of green tea extracts on the body systems in general and the nervous system particularly. For this, we orally administered lead, green tea, and a combination of lead and green tea to the experimental mice for 6 weeks. We found that the use of green tea restored the body weight gain initially induced by lead intoxication. Previous reports have also shown that lead exposure results in dose-specific weight gain in adult Westar rats [23] and that green tea has positive effects on weight maintenance [24]. Moreover, we noticed that supplementation with green tea has a positive effect on the hematological parameters in lead-intoxicated animals. Previous studies also reported the hematological changes induced by lead intoxication in albino mice [1,25]. It was found that RBCs have a high affinity for lead and approximately 99% of the lead present in the blood is bound to erythrocytes, which makes them more vulnerable to oxidative damage than many other cells [26].

Further, we observed the blood-glucose-lowering effects of green tea on the lead intoxicated group of mice. Previous data also suggest that green tea has an antidiabetic effect both in animal models and even in humans [12]. Interestingly, polysaccharide conjugates from the cell wall of green tea leave exert hypoglycemic effects by augmented insulin secretion. These polysaccharides are also reported to fine-tune the impaired glucose tolerance in vivo [27]. Green tea not only affects glucose metabolism, but also regulates lipid metabolism. Recently, the effects of various crude extracts of green tea on hepatic lipid metabolism and lipid profile have been investigated in mice and rats, respectively [28,29]. Our results further support these observations as we reported ameliorating effect of green tea on lead-associated dyslipidemia.

Our data display higher levels of lead in the blood and brain of lead intoxicated mice groups, which corresponds to many previous studies reporting similar results [30,31]. However, here, green tea supplementation exerted an obvious positive effect on the blood and brain levels of lead. Besides, in an earlier investigation, a mild lead exposure produced severe neurotoxic effects in rats [9]. Generally, lead mimics the calcium dependent pathways because of their similar charge and size. Lead binds to the calcium channels, thus substituting all calcium-dependent processes leading to disruption of brain and behavior [32]. Nevertheless, the protective effects of green tea extract on lead-induced oxidative and DNA damage in rat brains have been well documented [15,18]. In our previous study, we reported the genoprotective and anti-apoptotic effects of green tea [33]. Our current study elucidates that green tea has neurobehavioral positive effects as represented by the locomotory, anxiety, memory, and learning behaviors in green tea supplemented mice. Here, the lead-induced deficits were markedly reversed by the green tea administration. Likewise, another study on old-aged Winstar rats revealed that green tea supplementation can reverse the age-related deficits in learning and memory processes by inhibiting acetylcholinesterase activity [34].

The observed neuroprotective effects of green tea may be attributed to its chemical composition. It contains many polyphenolic compounds, generally known as flavanols or catechins [35] which are effective scavengers of superoxide, hydroxyl, and peroxyl radicals. In addition, catechins are known to chelate metal ions, another contributory mechanism in their protective action against the destructive effect of free radicals on the components of the blood cells [36]. Many studies reported the presence of (-)-epigallocatechin-3-gallate (EGCG), a gallate form of phenolics, in green tea extracts as the most potent and abundant compound in green tea. It possesses anti-proliferative, anti-mutagenic, antioxidant, antibacterial, antiviral, and chemopreventive effects [37,38]. Moreover, EGCG from green tea provides neuroprotection against β-amyloid-induced neuronal death. This protective mechanism often involves the inhibition of overproduction of reactive oxygen species (ROS) by EGCG in a dose-dependent manner [39,40]. Similarly, ECGC protected the Alzheimer-like rat model from neurodegeneration and oxidative damage in their hippocampus [41].

In the light of these findings, we analyzed the aqueous and ethanolic extracts of green tea to know the active ingredients that are responsible for its therapeutic effects. Our findings have revealed two important components which are caffeine and 1,2-benzenedicarboxylic acid, mono(2-ethylhexyl) ester besides other compounds with variable levels. The primary documented effects of caffeine are improvement of memory, concentration, and physical performance [42]. A recent report suggests that moderate and regular consumption of caffeine, a hydrophobic agent, protects against seizure and spatial memory loss [43]. Caffeine can easily cross the BBB, thus providing better neuroprotection against cognitive dysfunction due to excellent bioavailability in the brain [40]. This can rationalize the positive neurological effects observed in our current study. On the other hand, 1,2-benzenedicarboxylic acid mono(2-ethylhexyl) ester has antioxidant and anti-inflammatory properties [44]. Additionally, it exhibited in vitro anticancer potential against liver (HepG2) cancer cells [45]. We did not find ECGC in both crude extracts, which may account for selective solubility of polyphenols in different solvent systems. Taken together, our current data clearly illustrates that green tea has an ameliorating effect on the lead toxicity in mice and hence could be considered as a candidate for human application, especially in lead-polluted areas.

## 4. Materials and Methods

### 4.1. Animals

Forty (40) adult male albino mice (*Mus musculus*) with an average weight of 20.2 ± 2.24 g (8–9 weeks’ age) were obtained from the animal house at the College of Pharmacy—King Saud University and maintained and monitored in a specific pathogen-free environment. All animal procedures were performed as described before [46]. All animals were allowed to acclimatize in plastic cages in a well-ventilated room for one week before the experiment. The animals were maintained under the standard laboratory conditions described earlier [46], fed a diet of standard commercial pellets, and given water ad libitum.

### 4.2. Green Tea Extract Preparation

Green tea leaves were purchased from the “Two Leaves” company (Naya Bazar, Pahari Dhira Delhi-110006, India). The crude extract was obtained through the method of cold extraction by taking 120 g of powder in 400 mL of 98% ethanol into a clean dried glass vessel for two weeks at room temperature with shaking. After maceration, the mixture was filtered utilizing Whatman filter paper no.1 with a pore size of 11 µm to separate the extract from the plant debris. The extract was concentrated initially by rotary evaporator at reduced pressure and finally by open air. Animals were daily dosed with the leaves extract of green tea for six weeks.

### 4.3. Lead Preparation and Dosing Schedule

Lead acetate (C_4_H_6_O_4_PbH_2_O) with a molecular weight of 379.33 as pure crystals was obtained from Riedel-De HaenAGSeelze-Hannover Germany. Each mouse received 0.5 gm/kg of the extract. This dosage for animals’ treatment was chosen based on previous LD_50_ calculations in our lab. Animals were divided into 4 groups (10 mice/group) as follows: Group (I) control group (distilled water); Group (II) green tea group (0.5 mg/kg); Group (III) lead group (0.2 mg/kg); Group (IV) green tea + lead group (green tea and lead with the same concentrations as taken by groups II and III). Lead acetate and green tea extracts were delivered to the animals through oral intubation.

### 4.4. Monitoring of Water Consumption and Body Weight Changes

For six weeks, daily water consumption was monitored for all animals in the four groups. The animals were weighed before the commencement of administration and in subsequent weeks during the experiment period.

### 4.5. Behavioral Studies

Ten animals from each group were used in the current study. For testing, the animals were brought into a room (25 °C) of dim red light reserved for that purpose. All experiments were carried in the early morning for 6 weeks. In all of the behavioral tests, we followed the methods of Ajarem et al. [23].

Locomotory Behavior: The testing was done as described previously [23]. Briefly, the mice are placed in the center of the wooden cage and allowed to freely move for 300 s whereas the number of squares crossed, number of rears, number of wall rears, number of cleanings, duration of locomotion, and duration of immobility are monitored.

Fear and Anxiety Testing in Plus Maze: The testing was done as described previously [23]. Briefly, the mice are placed in the center of the maze and allowed to freely move for 300 s, whereas the number of entries into a closed arm, the number of entries into the open arm, the number of entries into the center, and the time spent for each one is monitored.

Learning and Memory Testing in Shuttle Box: Using the UGO BASILE shuttle box (Italy), whereas the mice are allowed to make thirty trials and the total latency time (La), the number of the reinforced crossing during the shock (Re), the number of intertrial crossing (Ic), the number of no crossing during the shock (Tr), and the number of the crossing during light/sound stimulus (St) are monitored.

### 4.6. Biochemical Assays

After six weeks, the mice were subjected to anesthesia by putting them in an anesthesia drop-chamber. Inside the chamber, a cotton gauze that was previously soaked with 2 mL of 20% isoflurane in propylene glycol was present. After that, cervical dislocation was done, and blood samples were collected from all animals, and plasma was separated. For cell blood count (CBC), heparinized blood samples were analyzed using Vet abc™ Animal Blood Counter (Horiba ABX, Montpellier, France). For glucose, cholesterol, triglycerides, and low-density lipoprotein (LDL) estimation; Reflotron^®®^ plus was utilized using 30-µL plasma samples being put in specific device strips.

### 4.7. Lead Estimation Assay

After completion of the behavioral experiment and blood collection, the mice were euthanized and their brain tissues were surgically removed, dissected, and preserved in normal saline. For the analytical determination of Pb, a 10% homogenate of the brain was prepared in Tris-HCl (50 mM). The homogenates were re-centrifuged at 17,000 rpm at 4 °C for 5 min and supernatants were filtered using 0.45 μm pore filters and analyzed by ICP–MS (Inductively Coupled Plasma Mass Spectrometer): ELAN 9000 (Perkin Elmer SciexInstrumento, Concord, Ontario, Canada). Nitric acid (69% *v/v*), super purity grade was supplied from Romil, Cambridge, UK. Hydrochloric acid (37% *v/v*) and hydrofluoric acid (40% *v/v*) were supplied by Merck (Darmstadt, Germany). High purity water obtained from the Millipore Milli-Q water purification system was used throughout the work. The ICP–MS calibration was carried out by external calibration with the blank solution and three working standard solutions (20, 40, and 60 ppm), starting from 1000 mg/L single standard solutions for ICP–MS (A ristar grade, BDH Laboratory Supplies, Poole, UK, for Cd).

### 4.8. GC-MS Instrumentation

Thermo Trace GC Ultra gas chromatograph coupled with TSQ Quantum mass spectrometer (triple quadrupole) (Thermo Scientific). The experimental conditions were optimized to set the main parameters of chromatographic separation, identification. GC conditions Temperature program: 50 °C (1 min), 10°/min, 250 °C (10 min), Split/splitless injector: 250 °C, Splitless mode, split flow: 50 mL/min, split time: 1 min, Carrier gas: helium, constant flow-rate: 1 mL/min, Transfer line temperature: 250 °C, Column Thermo Trace TR-5MS, Length: 30 m, i.d.: 0.25 mm, film thickness: 0.25 µm, Software: XCalibr software version 2.1, Mass Spectrometry (MS) conditions: full scan, mass range: 40–400 Da, scan time: 0.132 s, ionization mode: electron impact at 70 eV, emission current: 50 µA, compound identification was based on comparison of their mass spectra to those of reference standards obtained from spectral libraries NIST Mass Spectral Libraries v2.1. (National Institute of Standards and Technology) and Wiley.

### 4.9. Statistical Analysis

Before further statistical analysis, the data were tested for normality using the Anderson–Darling test, as well as for homogeneity variances. The data were normally distributed and is expressed as the mean ± standard error of the mean (SEM). Significant differences among the groups were analyzed by one- or two-way ANOVA followed by Tukey’s post-test using SPSS software, version 17. Differences were considered statistically significant at *p* < 0.05.

## 5. Conclusions

This study adds further evidence of the protective effects of green tea supplementation in lead-induced mice. Oral administration of green tea extracts improved the lead-associated pathological changes in the biochemical and neurobehavioral responses of treated mice in a significant manner. However, further studies are needed to better understand the underlying molecular mechanism for the neuroprotective effects of green tea.

## Figures and Tables

**Table 1:** Effect of green tea supplementation on the body weight and water consumption in the lead-intoxicated mice.

Groups	Body wt. (g)	Water Consumption (mL)
Control	34.30 ± 1.72	13.75 ± 0.15
Green tea group (GTE)	33.29 ± 2.42	14.49 ± 0.34
Lead group (Pb)	37.9 ± 1.07 *	10.02 ± 0.17 *
Lead + GT group (GTE + Pb)	35.19 ± 1.14	12.10 ± 0.16

Mean ± SEM., n = 10. * *p* < 0.05 for lead intoxicated group vs. control.

**Table 2:** Effect of green tea supplementation on the cell blood count (CBC) of mice.

Parameters	Control	Green Tea Group	Lead Group	Lead + GT Group
RBCs	7.73 ± 0.13	8.10 ± 0.15	8.45 ± 0.24	8.16 ± 0.33
WBCs	7.01 ± 0.22	6.36 ± 1.20	9.34 ± 1.55 *	8.03 ± 1.28
Hb	14.02 ± 0.51	15.08 ± 0.21	12.58 ± 0.35	13.74 ± 0.60
HCT	43.92 ± 1.65	47.66 ± 0.49 #	49.60 ± 1.33 *	43.86 ± 1.62
MCV	56.08 ± 2.11	59.00 ± 0.95	58.80 ± 0.86	53.80 ± 0.66
MCH	18.16 ± 0.62	18.62 ± 0.31	18.44 ± 0.39	16.84 ± 0.21
MCHC	31.96 ± 0.28	31.66 ± 0.14	31.42 ± 0.26	31.32 ± 0.22
PLT	657.8 ± 17.9	663.6 ± 19.2	793 ± 78.37 *	685.6 ± 23.8 &

Mean ± SEM., n = 10. * *p* < 0.05 for lead intoxicated group vs. control; # *p* < 0.05 for green tea group vs. control; & *p* < 0.05 for lead +GT group vs. control.

**Table 3:** Locomotory behavior testing in the experimental groups in the open area. * *p* < 0.05 for lead group vs. control.

Test	Control	Green Tea Group	Lead Group	Lead + GT Group
No. of squares-crossed	385	393	297 *	358 &
No. of rears	19	21	12 *	18
No. of wall-rears	38	40	24 *	33 &+
No. of washings	7	6	10 *	8
Locomotion duration (s)	190.6	197.7	104.8 *	139.4 &+
Immobility duration	110.5	103.9	198 *	121.3 &+

Values are Mean ± SEM., *n* = 10 * *p* < 0.05 for lead group vs. green tea group. & *p* < 0.05 for lead +GT group vs. control group. + *p* < 0.05 for lead +GT group vs. green tea group.

**Table 4:** Anxiety in plus-maze.

Parameter	Control	Green Tea Group	Lead Group	Lead + GT Group Pb
No. of entries into Open arm	7.3 ± 1.71	9 ± 0.92	6.8 ± 0.64	7.1 ± 0.81
Time spent in the open arm (s)	170 ± 0.28	173 ± 0.19	60 ± 0.2 *#	129 ± 0.19 &+
No. of entries into closed arm	4.9 ± 0.87	4.5 ± 1.09	6.5 ± 0.66 *#	5.6 ± 1.78
Time spent in the closed arm (s)	85.8 ± 0.56	83 ± 0.24	150 ± 0.23 *#	110 ± 0.27 &+
Time spent on the maze center (s)	40 ± 0.47	38 ± 0.34	57 ± 0.29 *#	48 ± 0.16 &+

Values are Mean ± SEM., *n* = 10. * *p* < 0.05 for lead group vs. control. # *p* < 0.05 for lead group vs. green tea group. & *p* < 0.05 for lead +GT group vs. control group. + *p* < 0.05 for lead +GT group vs. green tea group.

**Table 5:** Learning and memory test in automatic reflex conditioner (shuttle box).

Test	Control	Green Tea Group	Lead Group	Lead + GT Group
La (s)	120 ± 1.22	100 ± 0.99	150 ± 1.45 *#	125 ± 1.69 +
Ic	60 ± 1.6	63 ± 1.76	35 ± 0.37 *#	49 ± 1.32 &+
St	2.7 ± 0.4	2.9 ± 0.45	0.3 ± 0.33 *#	2.3 ± 0.26
Re	21.8 ± 2.41	21 ± 0.47	20.4 ± 1.76	20.7± 0.96
Tr	5.5 ± 2.45	6.1 ± 0.37	9.3 ± 1.9 *#	7.1 ± 0.99 &

Values are mean ± SEM., *n* = 10. * *p* < 0.05 for lead group vs. control. # *p* < 0.05 for lead group vs. green tea group. & *p* < 0.05 for lead +GT group vs. control group. + *p* < 0.05 for lead +GT group vs. green tea group.

## Data Availability

The datasets used and/or analyzed during the current study are available from the corresponding author on reasonable request.

## Sample Availability

Samples of the plant extracts are available from the authors.
